# The Aristotelian conception of habit and its contribution to human neuroscience

**DOI:** 10.3389/fnhum.2014.00883

**Published:** 2014-11-03

**Authors:** Javier Bernacer, Jose Ignacio Murillo

**Affiliations:** Mind-Brain Group, Institute for Culture and Society, University of NavarraPamplona, Navarra, Spain

**Keywords:** goal-directed actions, Aristotle, basal ganglia, cognitive control, prefrontal cortex, implicit memory, procedural learning

## Abstract

The notion of habit used in neuroscience is an inheritance from a particular theoretical origin, whose main source is William James. Thus, habits have been characterized as rigid, automatic, unconscious, and opposed to goal-directed actions. This analysis leaves unexplained several aspects of human behavior and cognition where habits are of great importance. We intend to demonstrate the utility that another philosophical conception of habit, the Aristotelian, may have for neuroscientific research. We first summarize the current notion of habit in neuroscience, its philosophical inspiration and the problems that arise from it, mostly centered on the sharp distinction between goal-directed actions and habitual behavior. We then introduce the Aristotelian view and we compare it with that of William James. For Aristotle, a habit is an acquired disposition to perform certain types of action. If this disposition involves an enhanced cognitive control of actions, it can be considered a “habit-as-learning”. The current view of habit in neuroscience, which lacks cognitive control and we term “habit-as-routine”, is also covered by the Aristotelian conception. He classifies habits into three categories: (1) theoretical, or the retention of learning understood as “knowing that *x* is so”; (2) behavioral, through which the agent achieves a rational control of emotion-permeated behavior (“knowing how to behave”); and (3) technical or learned skills (“knowing how to make or to do”). Finally, we propose new areas of research where this “novel” conception of habit could serve as a framework concept, from the cognitive enrichment of actions to the role of habits in pathological conditions. In all, this contribution may shed light on the understanding of habits as an important feature of human action. Habits, viewed as a cognitive enrichment of behavior, are a crucial resource for understanding human learning and behavioral plasticity.

## Introduction

In order to achieve a deep understanding of the main topics concerning the human mind, neuroscience must dialog with other sources of knowledge. In addition, from time to time, it is necessary to take a break from experimental work and ponder whether certain things taken for granted need to be revisited. Such is the case, in our opinion, with the concept of habit and habit learning. This revisiting has been profitably carried out in previous approaches to other topics, such as the self (Northoff, 2012).

In general terms, on the basis of experimental research in neuroscience, a habit is defined as a motor or cognitive routine that, once it is triggered, completes itself without conscious supervision. Furthermore, it has always been characterized via terms such as “unconscious”, “rigid”, “automatic” and, more importantly, “non-teleological”: that is, as the opposite of goal-directed. However, the original and most elegant description of habits, which goes back to Aristotle, defines them as acquired dispositions that improve the agent’s performance, making him/her more successful in the quest to achieve a goal. The neuropsychological distinction between goal-directed actions and habits (Dickinson, 1985) is, therefore, hardly compatible with this perspective. This distinction is based on two key phenomena: some behavior is habitual if it is performed even after (1) outcome devaluation or (2) a degradation of the action-outcome contingency. In other words, “habitual” behavior according to neuroscience is defined by the absence of self-proposed goals and a lack of cognitive control. These two elements, however, are crucial in the Aristotelian conception of habit.

This article will review, very briefly, the mainstream view of habit in neuroscience, its philosophical inspiration, as well as the challenges that recent research projects are encountering due to their reliance on this definition. We propose a multidisciplinary revised version of the notion of habit based on Aristotle’s work, and we explain to what extent it may help neuroscientific research. Finally, we suggest certain novel approaches to experimental research on habits, in order to attain a deeper understanding of the human mind.

In this article, our main purpose is not just to expose a terminological confusion that exists between neuroscience and philosophy. In fact, the common view of habit in neuroscience derives from a more specific view, which has its own history (Barandiaran and Di Paolo, 2014; Blanco, 2014). In our opinion, the notion of habit drawn from classical philosophy allows a better understanding of learning, including the role of routines and automatisms, in human behavior. We also believe that a richer view of habits in neuroscience may provide an improved interpretation of such “dichotomies” as conscious-unconscious, automatic-controlled, or teleological-ateleological, and may ultimately help to demonstrate that, in the case of human beings, these are not black and white processes employing binary variables, but arise from the complex interplay that configures human action.

## Current view of habits in neuroscience and its theoretical inspiration

An extensive review of the notion of habit in neuroscience is beyond the scope of this article, and we refer the reader to the works and reviews cited below for further reading. However, we summarize in a few paragraphs the conceptual background where habits reside in neuroscience, mainly based on the works by Anthony Dickinson and Ann Graybiel. The explicit investigation of habits in neuroscience is quite recent. This is a remarkable issue, if we accept that “we act according to our habits, from the time we rise and go through our morning routines until we fall asleep after evening routines” (Graybiel, 2008). All throughout the twentieth century, research on habits has centered on animal research, specifically on how behavioral patterns, i.e., motor routines, are developed and executed in non-human animals (see, for a historical review, the article by Seger and Spiering, 2011). One of the most important topics when studying habits in the field of neuroscience has been the relationship between actions, habits and goals. In that sense, the work by Dickinson (1985) was the seminal contribution. In his work, habits are overtly opposed to teleological actions, and identified with stimulus-response pairings. The main difference between these two processes is that, whereas actions are outcome-oriented and thus sensitive to reward devaluation or extinction, habits are just guided by the stimulus itself, and not by the outcome it leads to (Adams and Dickinson, 1981). Thus, a behavior is considered a habit when the animal insists on its performance in spite of outcome devaluation, or of the degradation of the contingency between the action and the outcome (Balleine and Dickinson, 1998; Yin and Knowlton, 2006). This is the mainstream view of habits in various subdisciplines within neuroscience, such as experimental psychology (Dickinson et al., 1998), psychiatry (Gillan et al., 2011), neuroanatomy (Yin and Knowlton, 2006) and neurocomputation (Balleine et al., 2008). Graybiel (2008) wraps this view up stating that habits are largely learned after extensive experience, remaining fixed and performed automatically, and they involve a structured action sequence triggered by a stimulus. Graybiel’s view on habits is not so clearly focused on the opposition between goal-directed actions and habits, although the defining characteristics of the latter, as proposed by her, are oriented towards those characteristics as defined by Dickinson. Goals are explicitly present during action evaluation and selection, but they increasingly blur the more an action is repeated. The main examples of habits Graybiel proposes are fixed action patterns, i.e., complex repetitive behaviors in animals, and repetitive behaviors and thoughts in human pathological conditions, such as Tourette syndrome, obsessive compulsive disorders, and stereotypies in Huntington’s and Parkinson’s diseases, as well as in addictive disorders. Therefore, a habit completely disengaged from a goal becomes either a stimulus-response pair in animals, or a pathological trait in humans. Graybiel also thinks that habits play an important role in societal terms, when they are shaped as mannerisms and rituals. However, the link between the anatomical and physiological bases of habits and their social expression is not clear at all, mostly because the majority of the experiments are carried out in laboratory animals. At a theoretical level, Graybiel describes an intuitive classification of habits as “neutral”, “good” or “bad”. Good habits would be those we strive to incorporate in our behavior, whereas bad habits are those that powerfully take control of our behavior. This categorization seems to leave a door open to include goals as drivers of habits: we interpret Graybiel’s “good” habits as those rationally directed to a goal, and “bad” habits as behavioral dispositions to perform rigidly defined actions uncontrolled by cognitive processes.

This is, very broadly, the current view of habits in neuroscience. In our opinion, it is of great interest to analyze the theoretical foundations of this conception, and to consider other alternatives that could enrich the study of habits in human neuroscience. As Seger and Spiering (2011) state in their recent review, the notion of habit in neuroscience is inspired by the view of the psychologist and philosopher William James (1890). James is also credited by Dickinson and Graybiel in their works. A succinct but clear explanation of the influences received by James in the formulation of his conception of habit has been recently published (Blanco, 2014), and we outline it here. According to James, habits can be innate or learned. In both cases they are based on plasticity, understood as a feature of inert matter, an idea taken from the French psychologist Léon Dumont: habit is just an analogy of the natural laws that affect the inanimate universe, but applied to living beings. Another important influence on William James’s idea of habit is that of William Benjamin Carpenter, an expert on comparative neurology whose conception of the unconscious inspired James. However, the main philosophical branch that influenced James was associationism, as understood by Alexander Bain and John Stuart Mill. This is the theoretical background that has had the greatest impact on the study of habit by neuroscience. The main idea is as follows: habits are based on the plasticity of matter, and they subserve adaptive purposes. Moreover, a habit can be chunked into smaller pieces that are automatically assembled: this is the main feature of associationism, and the start point of the Pavlovian stimulus-response pairing.

Another recent publication gives an extremely interesting genealogy of the concept of habit over the course of the history of philosophy and neuroscience (Barandiaran and Di Paolo, 2014). These authors state that “neuroscientific research on habit remains rooted within a narrow theoretical tradition”. Interestingly for the purpose of our manuscript, Barandiaran and Di Paolo (2014) acknowledge that the first description of habits was developed by Aristotle, and further interpretations of his findings have given rise to two opposed branches: organicism and associationism. According to these authors, the latter is the only theoretical influence in the study of habits in neuroscience. It is based on the idea that mental states are formed by the association of simpler units. Furthermore, the probability of one unit’s occurrence automatically following another’s increases when they have been contiguously presented in the past. The organicist view, although it has its origin in Aristotle, has been developed on the basis of the conception of *conatus* introduced by Spinoza: *conatus* is the essence of any “finite mode” (let’s say, as an example, any natural being), and it refers to the struggle to keep being oneself. Habits, then, are intended to preserve homeostasis of the organism, a view that differs from the original Aristotelian view, since according to the Greek philosopher good habits imply an increasing improvement of the agent. Going back to the organicist view, a habit includes the organism as a whole and its environment. The main difference between the associationist and the organicist interpretations of habits is, therefore, that the former views habits as an assembly of small mechanisms, whereas the latter considers them to be a resource of the organism—an embodied mind in a complex environment—that works to maintain homeostasis. Barandiaran and Di Paolo place William James in the associationist branch of the theoretical conceptualization of habit. The associationist heritage of James is clear in Chapter 4 (“Habit”) of “The Principles of Psychology”. Habits are based on the plasticity of brain matter and are characterized by the sequential functioning of different brain regions: “a simple habit, as every other nervous event (…) is, mechanically, nothing but a reflex discharge. (…) The most complex habits, (…) are, from the same point of view, nothing but concatenated discharges in the nerve-centers”.

At this point, we believe it may be useful to move back and forth between William James and the current notion of habit in neuroscience, in order to understand their similarities and differences. One of the results of James’s research is that “habit diminishes the conscious attention with which our acts are performed”. When we are learning a skill, “we interrupt ourselves at every step by unnecessary movements and false notes. When we are proficient, on the contrary, the results not only follow with the very minimum of muscular action requisite to bring them forth, they also follow from a single instantaneous ‘cue’”. This is the conceptual origin of the physiological chunking proposed by Graybiel and others (Graybiel, 1998; Barnes et al., 2005; Seger, 2008): when an animal is learning a motor sequence *de novo*, there is a continuous activation of the projection neurons located in the sensorimotor striatum; however, when the sequence is well-learned, these cells are significantly activated just in certain landmarks of the task, such as at the beginning and the end of the sequence. The decline in conscious attention suggested by James is also supported by current neuroscience, since the consolidated chunked activity in the motor aspects of the striatum during the performance of a well-learned motor sequence correlates with a decreased activity in the cognitive part of this brain area (Smith and Graybiel, 2014; Thorn and Graybiel, 2014).

We have outlined here the influence of William James on the current notion of habit in neuroscience, although much more could be said about this topic. The main conclusions of this initial part of our research are as follows: (1) most neuroscientists working on habits overtly credit the influence of James in their research; (2) James’s proposal is based on associationism, that is, small units that mechanically follow each other; (3) this association is the theoretical inspiration for the physiological chunking proposed as the neural bases of habits; and (4) having this theoretical background, experimental neuroscience has set the following condition for an action to be considered a habit: its performance must remain unchanged in the face of outcome devaluation and degradation of action-outcome contingency.

## Limitations

A notion of habit based solely on William James’s thought entails obvious benefits: neuroscientific research has achieved major advances in the study of the neurobiological foundations of motor routines, the relation of consciousness with habits, the mechanisms of instrumental learning in animals and the implication of these phenomena in human disorders, for example. However, this view is also limited to some extent, and we suggest overcoming these limitations with a different theoretical interpretation of habits.

The main shortcoming is that the opposition between goal-directed actions and habits—founded on the associationist view and developed on the basis of excellent animal research (Dickinson, 1985)—works experimentally, but it is far from explaining the complexity of human habits. This opposition has the strong point of being impeccable from an experimental point of view: a goal-directed action is driven by the outcome it leads to, whereas a habit is carried out even in the case of outcome devaluation or degradation of the action-outcome contingency. Therefore, experimentally and by definition, there cannot be goal-directed habits. However, this is not what we observe in human behavior, where many habits, even the simplest ones, such as tying one’s shoelaces, are goal-directed. As we will explain later, the fact of being or not being goal-directed is not necessarily the critical issue for distinguishing non-habitual from habitual behavior. It is interesting to note that the identification of habits as ateleological behavior is an interpretation of James’s work, although he himself does not put it in those terms. In fact, the first conclusion of his analysis is that “habits simplify the movements required to achieve a given result”. Thus, habits can be oriented towards a goal. When giving examples of human habits, he tended to mention musical performance, although the current notion in neuroscience is based more intensely on his “negative” examples, generally termed “slips-of-action”: once a behavioral sequence is initiated it can continue even beyond the intention that has elicited it: “Who is there that has never wound up his watch on taking off his waistcoat in the daytime, or taken his latchkey out on arriving at the door-step of a friend?”. Slips-of-action have been studied in the context of obsessive-compulsive disorder, where patients’ performance seems to be ateleological and, as some authors see it, “habitual”. Undoubtedly, acquiring a habit implies some determination; however, it is also worth noting that James leaves a door open to the conscious control of habits, since they “immediately call our attention if they go wrong”. Although we will not discuss the issue here for the sake of brevity, recent neuroscience research works accept that goals and habits are not strangers to each other: they can be intertwined in various ways, and not just during habit acquisition (Wood and Neal, 2007; Dezfouli and Balleine, 2013; Duncan, 2013).

Seger and Spiering uncover more limitations of this narrow view of habits, although they do not question the theoretical background that underlies them. When referring to habits and habit learning, the common interpretation of these phenomena employs several dichotomies to clearly distinguish them from goal-directed actions: rigid/flexible, slow/fast, unconscious/conscious, automatic/controlled, insensitive/sensitive to outcome revaluation (Seger and Spiering, 2011). At this point, we want to stress that these authors challenge a restrictive view of these “defining” characteristics of habits: (1) action categorization in the basal ganglia makes it possible to deal with new stimuli as if they were well-learned, allowing for some flexibility; (2) it is not clear how many trials are necessary for an “action” to become a “habit”, and for reaching a behavioral asymptote; (3) the various aspects of the basal ganglia (associative, sensorimotor and limbic) seem to be involved in conscious and unconscious learning, the distinction between which is far from being sharp (Horga and Maia, 2012); (4) automaticity has usually been assessed by dual-task performance, which is not actually an exclusive indicator of automaticity; and (5) outcome revaluation is a straight-forward method to be used in laboratory animals, but not quite so in humans.

In our opinion, the main problem with applying these categories to human behavior comes from the direct extrapolation of animal experiments to human research. Human cognitive resources are clearly different from those in animals. An inflexible comparison between the results of animal and human research would only shed light on the lowest levels of human cognition. If we focus our research strictly on those habits that animals are able to perform, or on those that fulfill the current theoretical model, we will constrain research on human neuroscience to investigating very simple habits. This interspecies correspondence could be also a consequence of the associationist heritage of the concept of habit, if the researcher assumes that the smaller units that constitute habits are the same in humans and non-human animals. Therefore the main limitation is, in our opinion, that habits are held as being apart from cognition, and this is why they are considered ateleological, rigid, unconscious, automatic and insensitive to outcomes. We next intend to demonstrate that the first conception of habit, found in classical Greek philosophy, incorporates cognitive control as a crucial element in its acquisition and performance, and that this theoretical framework may help in overcoming the limitations posed by the associationist view of habits in neuroscience.

## The original definition of habit

As we have seen, the dominant vision of habits in neuroscience conceives of them as a routine, very similar to the releasing mechanism that ethologists employ to analyze instinct (Tinbergen, 1951). The main difference between the two is that habits are not innate but acquired. After acquisition, they are considered to behave in a similar way as instincts: inflexibly, automatically and unconsciously.

However, this is not the first characterization of habit, historically speaking. The pioneering definition and analysis of habits were carried out by Aristotle, whose view has the great advantage of not being conditioned by the sharp distinction between conscious and unconscious processes, a dichotomy which is frequent in modern and contemporary thought. He explains his conception of habit in his book *Nicomachean Ethics* (Aristotle, 2002). Our analysis is based on the original version in ancient Greek, although we will cite versions translated into English for clarity. We have also freely translated some terms to show their similarity with concepts currently used in neuroscience, as we explain below.

According to the Aristotelian view, acquired habits presuppose behavioral plasticity, so that the agent can acquire new patterns of behavior in order to achieve a desired adaptation and so be more successful in the pursuit of his/her goals. Aristotle characterizes habits as dispositions, that is, particular arrangements of human capacities. The cornerstone that underlies the Aristotelian theory of action is the following: when an agent does or makes something, there is an effect not only on the receiver of the action or the product made, but also—and more importantly—on the agent. This is mainly explained in Book IX, chapter 8, 1050b 23–38 of *Metaphysics* (Aristotle, 2007) and in Book 3, chapter 7, 431a 5–9 of *On the soul* (Aristotle, 1986). Since human actions are driven and controlled by cognition, each new action leaves a footprint in the agent as a kind of learning: a disposition to face further similar situations in a certain way, which includes the interpretation of that situation and the possible ways of dealing with it. In some types of learning, this disposition also includes affective control and corporal skills. Please note that although Aristotle is highly subtle in his analysis, his conclusions are plain: one acquires a new ability by doing or making things related to that ability. As he states in Book 2 of the *Nicomachean Ethics*: “for the things we have to learn before we can do them, we learn by doing them, e.g., men become builders by building and lyre players by playing the lyre; so too we become just by doing just acts, temperate by doing temperate acts, brave by doing brave acts” (Aristotle, 2002). We add: through our actions, we acquire the disposition or habit of being builders, mathematicians, piano players or temperate. Since these habits are gained through practice, this process is goal directed.

In these first paragraphs we have outlined the Aristotelian view on habits, their place in his philosophical system and their characterization as learned dispositions. Next, we will show the ability of this conception to account for “good” and “bad” habits. As we mentioned above, this categorization has been suggested by Graybiel, who considers good habits as being those which we try to incorporate in our behavior, and bad habits as those that take control of our behavior (Graybiel, 2008). If we consider a habit to be a mere motor routine (or a behavior that remains unchanged after outcome devaluation or degradation of action-outcome contingency), it is hard to categorize it as good or bad in itself, because this usually depends on the context in which it is triggered. In our opinion, there is a key factor involved in considering habits as good or bad, appropriate or inappropriate: cognitive control. Through it, the agent can direct his or her behavior more adequately to the goal. If the acquisition of a habit implies a better cognitive control of the actions related to that habit, it can be considered as “good”. Otherwise, if it involves rigidity and blurs the goal, it is a “bad” habit. Since “good” and “bad” (or the Aristotelian terms “virtue” and “vice”) may sound odd to the neuroscientific community, we will term them “habits-as-learning” and “habits-as-routines”, thus highlighting the behavioral plasticity or the rigidity they lead to. In Book V of Aristotle’s *Metaphysics*, he states that “‘habit’ means a disposition according to which that which is disposed is either well or ill disposed, and either in itself or with reference to something else” (Aristotle, 2007). This, in our opinion, links habits to cognitive control and goals. Please note that the usual view of habit in neuroscience, inherited from associationism, corresponds to that subtype of habit that we have termed habits-as-routine. Therefore, habits-as-routines could be considered a cognitively-impoverished type of habits-as-learning. This is not surprising if we consider that such view has been elaborated on the basis of animal experiments, whose cognitive abilities are very limited in comparison with adult humans. We will elaborate on this point in the next section of our article, in order to demonstrate how the Aristotelian view gives an account of habits as motor routines, addictions and slips-of-action.

The main reason why Aristotle analyzed the nature of habits was to focus on ethics. From his point of view, ethics imply a broad study of human behavior (please note that “ethics” derives from “ethos”, which means conduct or behavior). However, the path he followed included a classification of acquired dispositions that can be of great interest for neuroscience. He distinguished three kinds of acquired habits, originally termed dianoethical, ethical and technical (Aristotle, 2002). In order to assist in a better understanding of the three types, we will use an updated terminology: theoretical, behavioral and technical. First, theoretical habits consist in the retention of learning. This is different from memory, the plain retention of former experiences. Theoretical habits are not acquired through mere experience and repetition, but require comprehension. A good example is the understanding of a mathematical discipline, like geometry, and the capacity to understand its internal coherence. A human being does not become a mathematician through simple repetition of operational routines; instead, he or she must understand mathematical concepts and theorems along with the deductions that prove them. Therefore, while comprehension is a key element of this type of habit, this is not the case for repetition: it depends on the quality of the action whether or not repetition improves the ability to understand the internal coherence of the discipline. Once acquired, a theoretical habit allows the agent to understand new concepts and propositions, and even to improve that particular discipline. This kind of habit is some sort of “know that”. In spite of the theoretical nature of these habits, they have a major influence on praxis because they allow cognitive abilities to develop. In neuroscience, this type of habit is usually studied as explicit memory (Schacter, 1987; Gabrieli et al., 1998), the “aha effect” (Luo et al., 2004)—the positive emotional response after understanding a concept or solving a problem—or the learning of a cognitive skill (Ashkenazi et al., 2013), for example.

The two remaining types of habits, however, improve behavior as well as the cognitive abilities that make it possible, rather than the theoretical abilities of the agent. In any case, Aristotle understands them as cognitive capacities as well, instead of mere routines. The second type is the behavioral habit, which depends on and is oriented towards *phronesis*: the habit of choosing and carrying out the best option for the agent in every situation. As Aristotle writes in the *Nicomachean Ethics*, “just as to practice medicine and healing consists not in applying or not applying the knife, in using or not using medicines, but in doing so in a certain way”. *Phronesis* is a Greek term usually translated as prudence, which is the perfection of practical reason. By exercising *phronesis*, the agent achieves a rational control of desires (*epithymia*: temperance) and impulses (*thymós*: fortitude). In turn, desires are another kind of behavioral habits connected to emotions. Hence, the key point here is that emotions can be rationally governed, and behavioral habits are the improvement of such control through qualified practice. *Phronesis*, or practical wisdom, also affects decision making by way of this adaptation of emotional responses to rationally proposed goals. Therefore, behavioral habits can be defined as knowing how to act; they are the basis of ethics and are studied in neuroscience under the umbrella of decision making (Caspers et al., 2011), moral judgments (Moll and de Oliveira-Souza, 2007) and in the context of the interplay between cognition and emotion (Pessoa, 2013).

The third type is technical habits, which include those learned skills of doing or making things qua directed to an external goal. They usually entail embodied skills, as in the case of playing a musical instrument, painting or competitive running. Motor routines, understood as habits by the associationist view and by neuroscience, would be included under the umbrella of technical habits, since in general technical habits involve the acquisition of psychomotor skills that, of course, are improved through practice. However, this third Aristotelian class of habits are not *just* habits-as-routines, since technical habits are also rationally controlled and, ultimately, goal-directed: knowing how to play the piano involves mastering certain motor skills, but also—and more importantly—putting them into practice in the right way and at the right moment. As Averroes—a philosopher of the 12th century and an expert on Aristotle—wrote, “habit is that whereby we act when we will”. This third kind of disposition consists therefore in knowing how to make or how to do. In neuroscience today, these habits are mainly analyzed as procedural learning (Censor et al., 2014; Pinho et al., 2014).

## Contributions of this definition of habit to neuroscience

This section intends to show why the Aristotelian theory of habits should be of interest for neuroscience. Before proceeding, we would like to clarify the main conclusions drawn from our analysis of the Aristotelian conception of habit presented in the previous chapter: (1) an acquired habit is an acquired disposition to perform certain types of actions; (2) this disposition, usually acquired by means of repetition of one or more actions, makes the execution of these actions prompter, more spontaneous and autonomous from continuous conscious supervision, all of which generally leads to a better performance; and (3) if the habit increases cognitive control of the actions, it can be termed a habit-as-learning; if on the contrary it increases their rigidity, it is a habit-as-routine.

As we mentioned above, the associationist view based on William James’s thought and introduced in experimental psychology has been extremely successful for understanding habits-as-routines in animals; however, it is quite limited when applied to human behavior, such as satisfactorily explaining good and bad habits, resolving the opposition between goal-directed actions and habits, overcoming the sharp distinction between conscious and unconscious processes or, more importantly, clarifying the role of cognitive control in human habits.

First, we would like to focus on the most important consequence of Aristotle’s research on this topic: habits contribute to the cognitive enrichment of actions. As in the case of the notion of habits-as-routines, all these capacities are acquired through a variable amount of practice: we become scientists through correct intellectual activity (Aristotle, 2002), and we improve the performance of a sequential finger motor routine through repetition (Nissen and Bullemer, 1987). However, habits-as-learning are not just the acquisition of a way of acting, but rather involve a cognitive capacity connected to the habit that can be flexibly utilized in different situations. As in the case of habits-as-routines, this capacity eliminates the need for fully conscious control of the basic components of the action in order to make possible the agent’s orientation to further and higher goals. For example, the pianist who can easily read the notes from the score (a mostly theoretical habit), and whose fingers appropriately respond to this reading (a technical habit) is able to exploit the expressive possibilities of the instrument. In summary, this feature of habits-as-learning is very important in order that this kind of habit may be read as a cognitive enrichment of behavior rather than as the acquisition of a routine. In the case of behavioral and technical habits they imply the availability of motor skills for complex activities, as well as the modulation of tendencies and desires to respond positively to conscious and rational goals. Therefore, they involve the acquisition of habits-as-routines, but their critical characteristics go beyond their motor aspects.

Second, another important difference between habits-as-routines and habits-as-learning is their differing relation to consciousness. For the former, habit performance is fully unconscious. In the latter, habits reduce or eliminate consciousness of basic elements of the action in order to concentrate on higher goals, while preserving at all times the possibility of recovering them for conscious attention. Although they seem unconscious and routinely performed, they are at the disposition of consciousness. They are not, in any case, rigid sequences. The possibility of developing habits-as-learning lies precisely in the feasibility of chunking those movements, actions and sequences, in order to organize them in other ways to perform different actions. Thus, pianists can learn how to play piano by repeating several motor routines, but they are not restricted to playing the routines they practice: their ability goes beyond those fixed movements to include improvisation. Therefore, the definition of habits-as-learning does not depend on the dichotomy of consciousness vs. unconsciousness. This is particularly important when this opposition is at stake in certain authors (Horga and Maia, 2012; Cleeremans, 2014).

The third contribution is two-fold: the Aristotelian view on habits allows us to understand the classification into good and bad habits, as well as to explain habits-as-routines (the notion of habit currently used in neuroscience) as a subtype of habits-as-learning. As we have outlined above, “good habits” can be defined as those that improve cognitive control, whereas “bad habits” are rigid behaviors nearly impossible to be cognitively regulated by the agent. This is intimately related to the interplay between habits and goals: since “good” habits involve an enhanced cognitive control, they lead us to a rationally proposed goal. In turn, this goal is enriched by the habit, as we explained in the case of the experienced pianist, who can concentrate on a better interpretation of the musical piece. On the other hand, the rigidity of “bad” habits leads us towards unwanted (non-rational) goals or away from rationally selected aims. Thus, addictions (Everitt and Robbins, 2005), compulsions (Gillan et al., 2011) and the susceptibility to slips-of-action (Norman, 1981) may be considered “bad” habits (or habits-as-routines) that could be due to a cognitive impoverishment of learned skills among other reasons. The acquisition of theoretical, behavioral and technical habits requires repetition; however, a high amount of it is not strictly necessary in the case of theoretical habits, since they can be acquired even by a single comprehension. In any case, it is important to emphasize that this repetition has to be qualified, rather than plain: the budding pianists have to have self-discipline in order to acquire habits that will help them to become virtuous. If they get used to performing the wrong movements, their ability will deteriorate. What is the critical feature that distinguishes a virtuous pianist from a regular piano player? It is, in our opinion, behavioral plasticity. If a student has acquired a set of cognitive-driven routines such that he or she can use them when they want to, it will result in a flexible performance. On the contrary, when routines have been learned through non-cognitive repetition, the final performance will be reduced to that set of routines.

This is also the case for behavioral habits: repetition of wrong behaviors causes the acquisition of habits with a poor or non-existent cognitive content. These are more similar to those routines that trigger “irrational” behaviors, such as addictions, compulsions and slips-of-actions. We would also include here unconscious biases that lead the agent to make a decision without considering all the relevant information (Kahneman, 2011). This would be a sort of “intellectual slip-of-action” that leads the agent to inadequately constrain the environment to be considered when making a decision. Rigidity is a consequence of the acquisition of habits that do not imply a cognitive enrichment of the action. Moreover, it is possible that some acquired skills may fall into rigidity and automation as a consequence of the decaying of higher cognitive functions, which by definition are in charge of controlling, reorganizing and reassessing acquired patterns. In fact, inappropriate habits imply a “negative” learning style that causes rigidity, as in the case of addiction or those technical habits-as-routines that cause difficulties with taking advantage of our possibilities—as in the case of the regular, but not virtuous, pianist.

Finally, the Aristotelian view on habits may provide new insights on the emotional response of the agent after habit acquisition. This is related, as we explain below, to the wanting/liking unbalance in drug addiction (Robinson and Berridge, 1993). Since cognitively controlled habits help the agent achieve rationally proposed goals, they tend to increase the enjoyment of the agent when performing such actions. However, the rigidity of habits-as-routines and the consequent blurring of goals diminish this enjoyment. Interestingly, this is in line with the current experimental approach to habits-as-routines in neuroscience: if the animal performs a goal-directed action, it has the pleasure of obtaining the reward; however, if its behavior has become a habit-as-routine, the “pleasure” is transferred to the response itself or to the cue that anticipates its performance. This results in an increased craving and a decreased pleasure after the outcome (Volkow et al., 2010).

## New perspectives for an interdisciplinary research

How can the Aristotelian notion of habit be of use in future research in neuroscience? In this last section, we would like to point to possible new directions for research on habits in human neuroscience. Whereas the successful experimental approach employing the associationist view of habits focuses on outcome devaluation and the degradation of the action-outcome contingency (Adams and Dickinson, 1981; Adams, 1982; Dickinson, 1985), we propose new criteria to be considered when researching human habits as a whole (both habits-as-learning and habits-as-routines). First, a habit will have been incorporated when its related actions are performed more spontaneously, that is, with greater promptitude. This could be quantified by a decrease in the reaction time of the deliberation prior to the action. Second, habit acquisition would imply a more accurate performance of the action, especially in the case of technical habits, measured by a decreased number of errors. Third, a categorization as habit-as-learning or habit-as-routine could be done by assessing cognitive control; behaviorally, this could be tested by error monitoring and adequately switching to a different task; neuroanatomically, by the recruitment of prefrontal regions and cognitive aspects of the basal ganglia. Finally, considering the relationship between the cognitive control of habits-as-learning and the enjoyment of their performance, a further indicator of their acquisition would be the recruitment of the reward system both before and after performance, whereas habits-as-routines would mainly involve the neuroanatomical “wanting” (incentive salience) system.

After these general experimental considerations, we focus on other topics within neuroscience where the dichotomy between habits-as-learning and habits-as-routines could be of great use.

### Habitual decision making

In a recent publication, we highlighted the difficulty of defining conscious (vs. unconscious) processes in “habitual decision making” (Bernacer et al., 2014). We are aware that this concept may sound provocative in neuroscience, since habits are related with unconscious phenomena, and decision making is mainly considered conscious, at least according to some accounts (Newell and Shanks, 2014). However, the nature of a decision should be considered with reference to its final goal. Driving is a technical habit that entails a high number of decisions, most of which are unconsciously made and performed: changing gear, putting the clutch in, switching on the indicator when turning, etc. However, driving is a conscious process overall: we decide to start the process, we consciously set the goal, and our driving is continuously available to conscious supervision. This framework is similar to the hierarchical model by Dezfouli and Balleine (2013), according to which habits are at the service of goal-directed behaviors. If we keep maintaining the extreme dichotomy between goal-directed actions and habits, we will be ruling most human activities out of the reach of neuroscience.

Furthermore, neuroscience can study the interplay between habits and decision making from another perspective. All three types of habits considered by Aristotle (theoretical, behavioral and technical) can be viewed as dispositions to configure one’s acting and, therefore, decision making. In recent years there have been a plethora of studies to determine the neural bases of human decision making (see, for example, the editor’s introduction to a special issue on this topic (Doya and Shadlen, 2012)). In short, it seems to be clear that the main players are the ventromedial prefrontal cortex (Levy and Glimcher, 2012), striatum and substantia nigra (Balleine et al., 2007). In addition, more dorsal aspects of the prefrontal cortex supervise the whole process (Manes et al., 2002), and other cortical regions are especially active in highly uncertain decisions (Hsu et al., 2005; Goñi et al., 2011). In a very simplistic—albeit accurate—way, humans decide to perform the action that carries the highest subjective value. This value depends on personal preferences, which in turn rely on the history of actions, decisions, skills and dispositions that the agent has carried out or acquired during his or her life. Thus, in many cases, decision making depends on habits. For example, it is well known that temporal discounting depends on personal preferences: people may tend to be either impulsive or else patient, and temporal discounting has been reported to correlate with the BOLD signal in the ventromedial prefrontal cortex (Kable and Glimcher, 2007). But, how do we initially become impulsive or patient? Is it encoded in our genes or in our neurotransmitters? Can our actions change this feature of our personality, as well as its in-brain correlate? In our opinion, the role of habits in decision making is a key topic for future research in cognitive neuroscience.

### Researching habits-as-learning in neuroscience

In the classical Aristotelian view, when the agent acquires a (good) habit, he or she performs the action: (1) more easily; (2) more efficiently; and (3) with higher enjoyment. This can be exemplified with the healthy habit of running: at the beginning, the jogger has to struggle to find the perfect time to go out, he or she can only run a short distance, and finds it definitely painful. However, as days go by, all three nuisances become increasingly tolerable.

Habits contribute to improving action performance because they release consciousness from having to focus on immediate goals, and allow all cognitive resources to focus instead on higher goals. This is the key idea for understanding how habits induce behavioral plasticity. A good pianist is able to improvise and concentrate on the artistic eloquence of the piece, because his or her acquired habit allows the player to go beyond the mere movements of his or her hands. This has been partially studied in neuroscience under the umbrella of “dual-tasking”, one of the measures of habits-as-routines. When a motor task is being learned, it requires the agent to expend a high amount of energy, since many executive brain areas are active; however, after practicing, brain activation is more restricted and energy consumption is thus lower (Poldrack et al., 2005). At the beginning, different aspects of the prefrontal cortex as well as their striatal targets—mainly the caudate nucleus—are in charge of the process; however, when the task is mastered, the activation of these areas is decreased and the putamen, globus pallidus and supplementary motor area of the cortex have a higher BOLD signal. This allows the prefrontal cortex and caudate nucleus to engage in a novel task when performing the well learned sequence.

This neuroanatomical framework is useful to understand those aspects of habits related with the automation of behavior. Automation is a condition for developing most habits-as-learning, since it releases executive areas from a continuous supervision of certain tasks. Thus, automations allow a cognitive enrichment of actions. It would be interesting to research, from the point of view of neuroscience, how this interplay between executive and “habit related” areas is carried out not only in motor routines, but also in more cognitive habits. For example, solving a Sudoku puzzle for the first time may seem overwhelming. With practice, the player discovers that as a result of performing certain intellectual routines the puzzle is easier to tackle. Furthermore, once these routines are acquired, it is easier for the player to monitor for errors and deal with new challenges within the puzzle. An area of possible future research is opened here, since error monitoring and problem solving will find their neural correlate in the prefrontal and anterior cingulate cortex; will, however, the intellectual routines be coded in the posterior putamen and premotor areas of the cortex?

Another interesting subject to investigate in the future is increased enjoyment in habit performance. Since this could be an extremely broad topic, we will only suggest its outlines here. For a start, it will be necessary to have an adequate characterization of pleasure and enjoyment. Human neuroscience assumes the reward circuit is an analog to that of non-human animals: unquestionably, regions such as the substantia nigra and the ventral striatum are active when an animal—rat, monkey or human—receives a primary reward (Schultz, 1997). It also happens when humans are granted a secondary reward, such as money (Delgado, 2007). However, human beings are also able to interpret as rewards things that are far from being pleasurable, including physically painful experiences. Thus, it may be appropriate to search for the brain correlates of these phenomena.

### Addictions, compulsions, stereotypies and slips-of-action: bad habits

Our article suggests a concept of habit that broadens the current view in neuroscience; for that reason, it has to be compatible with that very view. In this section we will briefly clarify how habits-as-learning can decay in humans to being habits-as-routines, leading thus to a behavioral rigidity of the agent, instead of flexibility. As we mentioned above, a habit turns into a mere automatic routine when its associated cognitive control declines. The role of cognition in habits is found first in goal setting. The initiation of a set of motor routines is meaningful if they serve a goal; otherwise, they can evolve into a compulsion or stereotypy. In his interesting review, Duncan (2013) cites the work of the Italian psychiatrist Bianchi (1922), who ablated different parts of the frontal lobe in monkeys to investigate changes in their behavior. Duncan highlights the following section of Bianchi’s work: “The monkey which used to jump on to the window-ledge, to call out to his companions, after the operation jumps on to the ledge again, but does not call out. The sight of the window determines the reflex of the jump, but the purpose is now lacking, for it is no longer represented in the focal point of consciousness... Evidently there are lacking all those other images that are necessary for the determination of a series of movements coordinated towards one end”. Therefore, the monkey can perform goal-directed motor habits while its frontal lobe is intact; when its cognitive function is damaged, the motor habits disengage from their goal and become a plain meaningless routine.

In any case, we believe that animal research on habits should be given due weight when transferred to humans, since the latter are able to acquire and perform habits that the former are not. The reason for this has been just outlined: cognitive control. The brain area involved in this phenomenon is the prefrontal cortex, which finds its greatest evolution in humans (Miller, 2000). Even though most of our knowledge on human neuroscience comes from animal studies, these are informative in the case of habits-as-routines, rather than habits-as-learning, because of the role of the cognitive enrichment of actions.

Finally, the lack of cognitive control in habits could be also a crucial feature in the case of compulsions in obsessive-compulsive disorder: washing one’s hands triggers a set of motor routines towards the goal of personal hygiene. However, when someone washes them repeatedly without a purpose, it becomes a compulsion. Slips-of-action may be understood as a temporary disengagement between a habit and its goal. They happen when the agent starts a goal-directed set of routines—for example, driving to a friend’s party—and ends up reaching an unwanted goal—arriving to the office instead of the party because the initial part of the driving routine is the same. In fact, these action errors have been related to obsessive-compulsive disorder (Gillan et al., 2011). Our proposal here, very briefly, is that routines could be incorporated in the agent—that is, coded into his/her brain, but would remain inhibited most of the time. Only when that routine needs to be executed do higher executive areas allow its performance through disinhibition. Again, in situations when cognitive control is compromised, the routine may be executed unwantedly (Mendez et al., 1997).

In sum, we believe that future research on stereotypies and compulsions should maintain its connection to the study of habits, but should focus on the lack of cognitive control rather than on the neural bases of the established motor routine, and bearing in mind that encouraging the patient to acquire cognitive-driven habits may help overcome rigid routines (Güell and Nuñez, 2014).

## Conclusion

Our interdisciplinary research seeks to provide a more adequate framework for the study of habits in neuroscience. We have demonstrated that this perspective is compatible with past and current experimental research, and it expands its scope when applied to humans. From a holistic view of human behavior, habits are very important aspects for behavioral plasticity and learning, since they release cognitive areas to focus on higher goals. Further, even though repetition and routines are important for habit acquisition, they can also be considered a crucial element for human behavioral freedom (Bernacer and Gimenez-Amaya, 2013), inasmuch as they increase the repertoire of actions and allow a better cognitive control of behavior.

Revisiting the ideas of classical philosophers may be very useful for neuroscience, since the cognitive and psychological substrate of neuroscience is composed, at least in a high proportion, of the ideas developed by philosophers across the centuries. We believe interdisciplinary research may help achieve a better understanding of human behavior, and provide the neuroscience community with an adequate theoretical background for undertaking new experimental approaches and tackling the challenges resulting from them.

## Conflict of interest statement

The authors declare that the research was conducted in the absence of any commercial or financial relationships that could be construed as a potential conflict of interest.
